# Loneliness and depression in older adults with multimorbidity: the role of self-efficacy and social support

**DOI:** 10.3389/fpsyt.2023.1232067

**Published:** 2023-10-30

**Authors:** Annika Roskoschinski, Wei Liang, Yanping Duan, Hayl Al-Salehi, Sonia Lippke

**Affiliations:** ^1^Unit for Geriatrics and Physical Medicine, Helios Klinikum Berlin-Buch, Berlin, Germany; ^2^Constructor University Bremen, Bremen, Germany; ^3^School of Physical Education, Shenzhen University, Shenzhen, China; ^4^Hong Kong Baptist University, Kowloon, Hong Kong SAR, China; ^5^Bremen International Graduate School of Social Sciences (BIGSSS), Bremen, Germany

**Keywords:** multimorbidity, older individuals, COVID-19, loneliness, self-efficacy, depression, social support, lifestyle

## Abstract

**Introduction:**

As relatively little is known about self-efficacy and social support in individuals aged 65 years and older and whether they are facing a decline in life due to multimorbidity and previous COVID-19 infection, this study investigated hypotheses based on Social Cognitive Theory.

**Methods:**

It was tested whether depressive symptoms in multimorbid patients who were hospitalized for COVID-19 infection, and recover post infection during their hospital stay, do not differ from those of multimorbid patients hospitalized for other conditions. Furthermore, we tested whether depressive symptoms are associated with increased loneliness scores, low self-efficacy beliefs, and poorly perceived social support. Additionally, it was investigated whether self-efficacy is a mediator variable, and social support is a moderator variable between loneliness and depression. *N* = 135 patients with or without previous COVID-19 infection (mean age 64.76) were recruited. Paper questionnaires were collected at the time of inpatient hospital admission in the year 2021 and in a cross-sectional study design. The study compared *n* = 45 multimorbid patients who survived COVID-19 infection with those *n* = 90 who were not infected before.

**Results:**

No significant difference in depressive symptomology between these two groups revealed [*t*_(133)_ = 130, *p* = 0.90, *d* = 0.024); *F*_(3, 122)_ = 0.255, *p* = 0.86]. The study found a positive correlation between loneliness and anxiety and depression in both groups (*r*_depression_ = 0.419 and *r*_anxiety_ = 0.496). Self-efficacy mediated the relation between loneliness and depression. The completely standardized indirect effect was *β* = 0.111, percentile Bootstrap 95% CI 0.027–0.201.

**Discussion:**

The research findings suggest the importance of self-efficacy, and loneliness in the development of depressive symptoms, and have several practical implications for improving the mental health of multimorbid patients: Prospectively, treatment should not only focus on physical and cognitive health, but also on promoting self-efficacy and perceived social support, as well as address loneliness with psychoeducational interventions. Replication of the findings and conducting interventional research also employing lifestyle components should follow up, as this study tested associations but no causal relationships.

## Introduction

The prevention and treatment of depression in elderly, multimorbid individuals in an age of approx. 65 years require a better understanding of various factors, especially those regarding the role of lifestyles. Lifestyle can be regarded as behaviors and general social-cognitive factors such as self-efficacy and social support (1). While there is already some evidence for behaviors such as caring activities and social activities (2), little is known about self-efficacy and social support as key components of social determinants of health (3) in individuals facing a decline in health with aging. Decline in later life was observed especially in hospitalized elderly multimorbid patients after COVID-19 infection but also in hospitalized multimorbid patients without known COVID-19 infection during the COVID-19 pandemic.

As reported in a literature review by Hossain et al. (4), multiple studies indicate mental health constraints in the general population, including anxiety and depression with varying levels of severity, since the outbreak of the COVID-19 pandemic in Wuhan, China, in late 2019. For example, in a study of 1,593 participants over 18 years of age in southern China, Lei et al. (5) reported that the prevalence of depression and anxiety among people who were directly or indirectly affected by COVID-19 through quarantine was 14.6 and 8.3%, compared with unaffected individuals (11.9%, 6.7%). Respondents were divided into two groups: those who were directly or indirectly affected by COVID-19 (for example, by quarantine due to their own COVID-19 infection or by infected family members, neighbours, or colleagues) and the second group who were not affected during the pandemic.

According to data by Huang et al. (6), 23% of all COVID-19-infected patients suffered from depression and anxiety after surviving a COVID-19 infection. Guo et al. (7), also reported that hospitalized patients infected with COVID had higher depression and anxiety scores than the comparison group of non-infected. Ismael et al. (8) reported in their prospective cohort study that there is a need for prospective studies to assess psychiatric symptoms in COVID-19 patients in the post-infection period. A total of (*N* = 895) who were tested for COVID-19 by nasopharyngeal swabbing and classified as mild cases were also tested for psychiatric symptoms. They concluded that during the course (approximately 2 months), an increased number of COVID-19 symptoms was associated with clinically significant increased scores for anxiety 22.4% (*n* = 201) and depression 26.2% (*n* = 235) and recommended monitoring the development of these symptoms after discharge from COVID-19 treatment. Kong et al. (9) investigated depression predictors and prevalence in acutely ill COVID-19 patients (*n* = 144). The risk factors that were specifically associated with depression in the Kong et al. (9) study were lack of social support and older age (>50 years). However, in this study, “elderly” people are referred to as individuals, at an age of approximately 65 years. According to Singh et al. (10), age 65 and older is commonly used as the cutoff for “elderly”. This criterion of 65 years is also used in the current OECD definition (11) and is also referred to in this study.

Coexisting chronic diseases are a risk factor for health anxiety in the COVID-19 pandemic in Turkey, as reported by Özdin et al. (12). Whether this is also true for elderly multimorbid post-COVID patients in Germany and whether other factors, such as feelings of loneliness or treatment (intensive care unit or normal ward), are related is still under investigated. The NIHR-National Institute for Health and Care Excellence (13) defines multimorbidity or ‘multiple long-term conditions’ according to (14) as the presence in an individual of two or more chronic conditions that are distinct from each other, where none of the individual conditions is an index condition.

Subjective decline and mental health issues such as depression can accompany or be triggered by physical illnesses, e.g., in hospitalized older multimorbid patients as it was found with individuals with chronic pain, patients with chronic obstructive pulmonary disease (COPD) or heart disease, as shown by data prior to the COVID pandemic (15–19). This raises the question of whether the symptomatology of depression is specific to post-COVID patients. Based on the previously described research results, it can be assumed that the increased depression scores in multimorbid patients after COVID-19 infection are not COVID-specific. In general, the following predictors for the development of depression have been described: female gender, somatic illness (20), loneliness (21), and lack of social support. High levels of social support were found to be a protective factor against depression (9, 22).

In contrast, low self-efficacy is associated with loneliness and depression (23). Self-efficacy and loneliness are critical indicators for developing depressive symptoms (21, 23). Self-efficacy is commonly associated with loneliness (24–26). Sierakowska and Doroszkiewicz (27) also recently reported significant associations between loneliness and generalized self-efficacy in the general population during the COVID-19 pandemic. Therefore, the constructs loneliness, self-efficacy beliefs, and social support are included as variables in this study.

Loneliness is a psychological construct that was increasingly often investigated in original studies, reviews, and meta-analyses (28). Cacioppo et al. (29) concluded that loneliness contributes to physical and psychological disorders such as depression. Loneliness can predict depressive symptoms (30, 31). Other studies confirm the link between loneliness and depression (21, 25) and the role of loneliness as a mediator variable between other psychological constructs (32). Stickley and Koyanagi (33) reported that the association between physical multimorbidity and loneliness was significantly mediated by depression (15.4%) and that the association between loneliness and multimorbidity remained largely unexplored. The association between multimorbidity and increased loneliness scores was also described by Kristensen et al. (34) and Hajek et al. (35).

The theoretical foundation of this study is Bandura’s (36) Social Cognitive Theory (SCT). This SCT is a further development of his self-efficacy theory (36–41). Bandura’s SCT describes factors that influence and determine behavior (39) and includes the following core constructs: perceived self-efficacy, outcome expectations, goals, and facilitators and barriers (42). The facilitators and barriers are social structural factors such as the participant’s environment, including cognitions about social support or the social environment. These factors interact with each other (37). The SCT assumes that self-efficacy is the result of an interplay of cognitive, motivational, behavioral, and also social abilities and is not a stable, hardly changeable character trait (38).

According to Bandura (37), social influences play an important role in the development of behavior that can subsequently impact health, as individuals are directly or indirectly influenced by the behaviors, actions, thoughts, and feelings of others. Based on SCT, McAuley et al. (43) revealed a strong correlation between the constructs self-efficacy and social support in their study. We elaborated on both factors in more detail in the following to investigate it in the current study.

Self-efficacy Theory by Bandura (40) states that self-efficacy is a mediator of various health outcomes. According to Tripathi and Asthana (25), a strong self-efficacy expectancy proved to be a protective factor and contributed to reduced depression and loneliness. Tsuji et al. (44) reported that the more pronounced a subject’s self-efficacy belief, the lower their depression scores. Conversely, self-efficacy expectancy was less pronounced in patients with elevated depression.

Self-efficacy is imperative for lifestyle (1), and as lifestyle as well as self-efficacy directly impact loneliness and depression (23, 26, 45–47) the mechanisms needs to be understood better especially at the beginning of treatment: previous mastery experiences and an optimistic view toward the future can be addressed especially if their levels are not optimal (28). With that, self-efficacy counts as a central lifestyle factor for adults’ mental and cognitive health. Kim et al. (45) showed that self-efficacy is significantly positively correlated with health-promoting behaviors in older women. When testing elderly participants in a moderated mediation, Lara et al. (46) found that the higher the level of self-efficacy, the better the mental health of those who perceived increased social support.

Self-efficacy as a predictor of loneliness or a negative correlation between the two constructs has been confirmed in several studies (25, 26). Peters et al. (47) showed that lower self-efficacy in individuals with multimorbidity decreases their quality of life. Nieboer et al. (48) examined the relationship between self-management skills, self-efficacy beliefs, and loneliness in elderly subjects, showing that self-efficacy is a protective factor against emotional loneliness. Lee et al. (49) also found a negative association between coping self-efficacy, the confidence to overcome challenges using social support, and loneliness in a study of 151 subjects aged 65 and older. They conducted a group comparison between subjects who felt lonely and subjects who did not and came to the following conclusions: the chronic disease symptoms was higher in the group of subjects feeling lonely than in the non-lonely control group. Similarly, the group of lonely subjects showed lower scores in coping self-efficacy as well as lower perceived social support. Depressive symptoms were also more pronounced in the lonely group. Conversely, higher coping self-efficacy and higher social support scores were associated with lower odds of loneliness. Holt-Lunstad et al. (50) report in a meta-analytic review that feeling both actual and perceived socially isolated is associated with an increased risk of early mortality, especially for individuals with an average age of less than 65 years. In a previous meta-analytic review, which included 148 independent studies, Holt-Lunstad et al. (51) showed that the probability of survival was increased by up to 50% in participants (mean age 63.9) with cardiac, neurological, or cancer diseases if they had strong social relationships.

Another lifestyle factor is social support because it relates not only to social activities (2) but also to mastery experience and functional social integration. Muhammad and Maurya (52) reported that older adults with functional impairments in daily living, *lack of social activities*, and living separately were more likely to develop symptoms of major depression. According to Chen et al. (53), increased social support in older adults is associated with reduced levels of loneliness and depression and reduced negative coping styles, i.e., attempts to forget past events, giving up to hopelessness, and indifference towards finding solutions to persisting personal problems. A comparative study by Olaya et al. (54) showed a significant interaction between social support and multimorbidity (*p* < 0.01). Patients with low social support and two chronic diseases had a lower survival probability compared to non-multimorbid patients. Kong et al. (9) reported that social support can lower depressive symptoms in acutely ill COVID-19 patients.

In contrast, depressive symptoms were shown to mediate between social support and quality of life among older adults in rural China, with direct positive correlation between quality of life and social support, while concurrently elevated depressive symptoms were typically associated with lower levels of social support (55). Liu et al. (56) reported that social support mediates between loneliness and depression. Golaszewski et al. (16) showed that social support inversely correlates with social isolation and loneliness.

According to the German *S3-Guideline Multimorbidity* (57), chronically ill and multimorbid patients require special attention as they often suffer from mental illnesses, especially depression, and anxiety, as well as they have an above-average mental burden compared to the general population. Clarke et al. (58) noted in their meta-analysis that there is robust evidence of the association between depression and physical illness. Bu et al. (59) confirmed that social support was a protective factor against loneliness during the first pandemic lockdown in the UK. Hsu and Chao (60) demonstrated that satisfaction with social support from family was a protective factor against loneliness.

This cross-sectional study aimed to explore the descriptive characteristics of multimorbid patients in terms of their mental status during hospitalization. In addition, this study aimed to examine associations and the relationship between loneliness and depression, as well as the mediating role of self-efficacy and moderating role of perceived social support in the aforementioned association in older adults with multimorbidity. It was hypothesized that:

Hypothesis 1: Depressive symptomatology is invariant in multimorbid patients after a COVID-19 infection compared to multimorbid patients without a known COVID-19 infection.

Hypothesis 2: Mental health constraints in multimorbid patients, including depression and anxiety symptomatology, are associated with increased loneliness scores, low self-efficacy beliefs, and insufficient perceived social support.

Hypothesis 3: Self-efficacy mediates the relationship between loneliness and depression.

**Figure 1:**
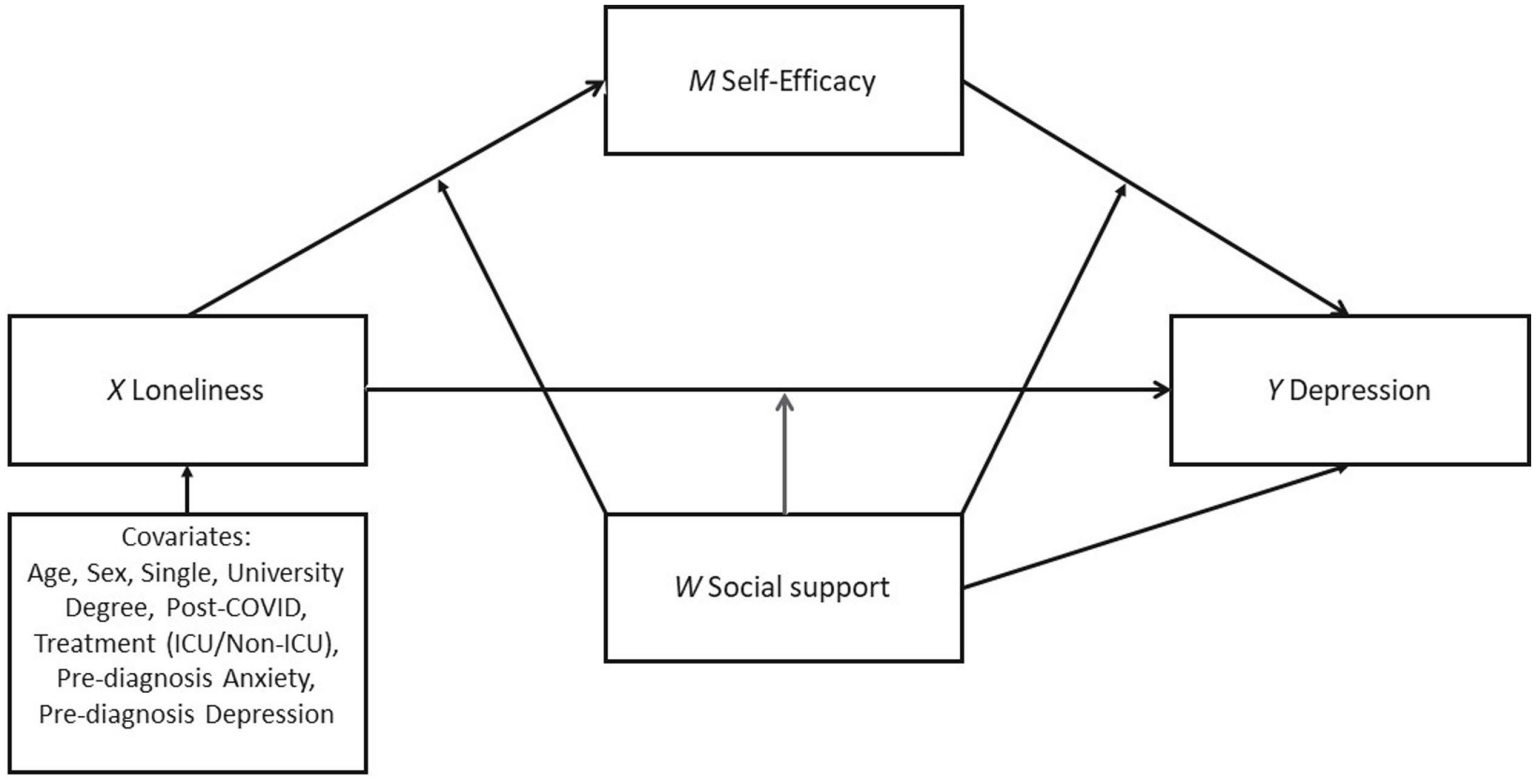
The assumed moderated mediation model predicting depression by loneliness with self-efficacy as mediator and social support as moderator.

Hypothesis 4: Perceived support plays a moderating role in the mediation model of loneliness, self-efficacy, and depression (see Figure 1).

## Methods

To test the hypotheses, a cross-sectional study with one measurement point was conducted to examine associations between the variables loneliness and depression as well as a mediating and moderating role of self-efficacy and social support.

### Participants

A total of (*n* = 135) patients (Table 1) were interviewed at the time of inpatient admission to a specialized clinic (T1). This particular sample was collected to ensure a robust estimate in the mediation model and to reach as many subjects as possible within this specific patient group. Empirical data were collected from the patients, including demographic information of the patients in addition to several measurements of psychological constructs to test the hypotheses. Participation in the study was voluntary and required informed consent. The approval of the Ethics Committee of Constructor University (formerly Jacobs University Bremen), Bremen, Germany, has been obtained (Application No: 2020_06). We assume a moderate correlation coefficient (*r*) and calculated with (*r* = 0.3) in the power analysis using G*Power 3.1.9.7 (61) (α = 0.05, 95% statistical power (1-β), two-tailed). Resulting from this calculation, 138 subjects should be included; however, the correlation coefficient *r* would remain moderate even if we included 135 subjects as further investigations with G*Power demonstrated. Thus, we aimed to recruit 135 study participants.

**Table 1:** Characteristics of the study sample (*N*=135).

	Post-COVID		Non-COVID		Total	
	N/M	%/SD	N/M	%/SD	N/M	%/SD
Total	45	33.3	90	66.7	135	100
Gender						
Female	20	44.4	43	47.8	63	46.7
Male	25	55.6	47	52.2	72	53.3
Age (years)	65.09	10.27	64.6	7.10	64.76	8.26
Education						
No formal education	1	2.2	1	1.1	2	1.5
8th grade graduation	8	17.8	9	10.0	17	12.6
10th grade graduation	6	13.3	9	10.0	15	11.1
Vocational training	11	24.4	42	46.7	53	39.3
Technical college degree/ master craftsman	7	15.6	16	17.8	23	17.0
A-Level (university-entrance diploma)	2	4.4	2	2.2	4	3.0
University degree	8	17.8	10	11.1	18	13.3
PhD/doctorate	1	2.2	-	-	1	0.7
Marital status						
Single	12	26.7	24	26.7	36	26.7
In partnership	3	6.7	8	8.9	11	8.1
Married	11	24.4	23	25.6	34	25.2
Divorced	14	31.1	20	22.2	34	25.2
Widowed	5	11.1	14	15.6	19	14.1
ICU Treatment	30	66.7	46	51.1	76	56.3
Non-invasive ventilation	31	68.9	47	52.2	78	57.8
Invasive ventilation w/coma	23	51.1	27	30.0	50	37.0
Outcomes variables						
UCLA loneliness scale	2.31	0.99	2.24	0.94	2.26	0.95
HADS-D depression	1.13	0.74	1.15	0.67	1.15	0.70
HADS-D anxiety	1.08	0.69	1.05	0.68	1.06	0.66
SWE – general self-efficacy expectancy	2.84	0.64	2.82	0.67	2.83	0.67
Perceived social support	3.88	0.97	3.82	1.02	3.84	1.00
Anxiety increased T1	20	44.4	44	48.9	64	47.4
Depression increased T1	23	51.1	43	47.8	66	48.9

HADS-D = hospital anxiety and depression scale (HADS-D, German adaptation); F-SozU K-6 = perceived social support questionnaire; SWE – general self-efficacy expectancy scale.

For mediation (2 predictors, 1 criterion) and moderation (α = 0.05, 95%, two-tailed) analyses, we assumed a medium effect of *f*^2^ 0.15 [Cohen’s f^2^: small ≥0.02; moderate ≥0.15; large ≥0.35, (62)]. According to G*Power, at least 89 subjects should be included, which we aimed for in our study. The moderated mediation model included 117 multimorbid patients. Eighteen patients (*n* = 18) were excluded due to incomplete questionnaire responses.

The sample was recruited from a physical medicine and geriatrics unit at the clinic Helios Klinikum Berlin-Buch, Berlin, Germany. Patients were recruited to the study by means of convenience sampling. To counteract selection bias, inclusion, and exclusion criteria were defined (see below). Patients who were multimorbid, previously diagnosed with COVID-19, and multimorbid patients without previous COVID-19 infection were recruited for the study. They stayed an average of two weeks, during which time data were collected and patients were screened for inclusion and exclusion criteria by their treating psychologists. Two groups were created for this purpose. Multimorbid patients in the first group who were previously in the COVID-19 unit for acute COVID-19 infection and then transferred to the unit for “Physical Medicine” after a COVID-19 infection for a so-called early rehabilitation to recover were surveyed. In the second group, multimorbid patients who were in early rehabilitation in the same unit for recovery from other conditions but who had no prior knowledgeable COVID-19 infection were surveyed. These were admitted as inpatients because, for, e.g., for further recovery after cardiological or gastrointestinal diseases, such as heart attack or colon cancer, neurological diseases (e.g., stroke) and other variety of conditions from other units or hospitals. Early rehabilitation is a form of treatment in the clinic for patients who, after acute illness, are not yet fit enough to be discharged home or to proper rehabilitation and still require further follow-up care and must continue to be under internistic observation.

The study excluded patients with high language barriers and those suffering from dyslexia, as well as those with severe intellectual or cognitive impairment (e.g., underlying psychiatric disorders, such as schizophrenia, dementia, or acute delirium). The survey was conducted between January 2021 and May 2022 at the clinic Helios Klinikum Berlin-Buch, the unit of Geriatrics and Physical Medicine. The characteristics of the recruited sample group are shown in Table 1.

### Measures

The following psychological attributes are measured: loneliness, depression, anxiety, subjectively perceived social support, and general self-efficacy beliefs. The following instruments were used for assessment: TILS [Three Items Loneliness Scale, Cronbach’s *α* = 0.72 (63), HADS-D – Hospital Anxiety and Depression Scale-German Version, HADS-D Cronbach’s *α* = 0.82, HADS-A Cronbach’s *α* = 0.83 (64), F-SozU K-6 – Perceived Social Support Questionnaire, Cronbach’s *α* = 0.90 (65), and the SWE-General Self-Efficacy Expectancy Scale, Cronbach’s *α* = 0.78 to 0.79 (66)]. The self-efficacy and social support scales are original German questionnaires that were used in this study. Likewise, a normed, validated, and reliable German version of the HADS was available and used accordingly. The 3-item version of the UCLA Loneliness Scale was also available in the German translations [Cihlar et al. (67); German Socio-Economic Panel study – SOEP-Core 2017 by Kantar Public (68)], which differ slightly grammatically but are the same in semantic meaning. In this study, the translation of Cihlar et al. (67) was used, which is as close as possible to the English original.

All participating multimorbid patients were asked to fill in the questionnaires shortly after their inpatient admission. Sociodemographic characteristics such as age, gender, relationship status (single 1/0), education (university degree 1/0), previous illnesses (F diagnoses of depression or anxiety according to ICD-10 (69)) were included in the survey. Also included was whether patients were treated in normal wards only, without prior treatment in the Intensive Care Unit, or whether treatment in the Intensive Care Unit also preceded treatment in the Physical Medicine Unit.

### Data analysis

To test the hypotheses of this study, statistical analyses were implemented using IBM SPSS 29 (Armonk, NY, United States). For hypothesis 1, a *t*-test was performed to examine whether depressive symptomatology was invariant between multimorbid non-COVID-19 and multimorbid post-COVID-19 patients (Table 2; Table A1). For hypothesis 2, intercorrelations were computed to answer the assumption of whether mental health problems in multimorbid patients, including depression and anxiety symptoms, may be associated with increased levels of loneliness, low self-efficacy beliefs, and insufficient perceived social support. For hypotheses 3 and 4, a moderated mediation analysis was conducted to test whether self-efficacy mediates in the relationship between loneliness and depression and whether social support has a moderating role in the relationship between these 3 variables.

**Table 2:** Comparison of the results of the variable scores in post-COVID patients and patients with non-COVID patients.

	Sample	*N*	*M*	SD	*p*	Cohen’s d
Loneliness	NON COV	90	2.24	0.94	0.695	−0.077
	POST COV	36	2.31	0.99		
Anxiety	NON COV	90	1.05	0.65	0.790	−0.049
	POST COV	45	1.08	0.69		
Depression	NON COV	90	1.15	0.68	0.897	0.024
	POST COV	45	1.13	0.74		
Self-efficacy	NON COV	89	2.82	0.67	0.820	−0.042
	POST COV	45	2.84	0.64		
Social support	NON COV	90	3.82	1.02	0.744	−0.060
	POST COV	45	3.88	0.97		

NON COV = multimorbid patients admitted to the hospital for other causes of treatment; POST COV = multimorbid patients with a previous COVID-19 infection.

Due to the significant correlations between the variables (Table 3), a moderated mediation analysis was conducted using PROCESS v4.1, Model 59 (74) to examine whether self-efficacy mediates the relationship between loneliness and depression and whether social support acts as a moderator variable. Parameters were estimated using the bias-correlated bootstrap approach (5,000 resamples). In addition, a purely mediation model that excluded the moderator was further examined as sensitivity test using PROCESS model 4 (74) in case of a prominent multicollinearity between studies variables. The statistical significance was set as *p* < 0.05 (two-tailed) for the data analysis.

**Table 3:** Variables, Sample items of the German questionnaire translated to English and results of the Pearson’s r correlation analysis (*n* = 125–135), 2-tailed.

Variables researched in this study	Sample item	*M*	SD	1.	2.	3.	4.	5.
1. Loneliness^1^	How often do you feel left out – (1) Never; (2) Hardly ever; (3) Some of the time; (4) Often	2.26	0.95	(0.85)				
2. Anxiety^2^	Worrying thoughts go through my mind – (3) A great deal of the time; (2) A lot of the time; (1) From time to time, but not too often; (0) Only occasionally	1.06	0.66	0.496^**^	(0.81)			
3. Depression^3^	I feel as if I am slowed down – (3) Nearly all the time; (2) Very often; (1) Sometimes; (0) Not at all	1.15	0.70	0.419^**^	0.635^**^	(0.82)		
4. Self-Efficacy^4^	I can always manage to solve difficult problems if I try hard enough (1) Not at all true; (2) Hardly true; (3) Moderately true; (4) Exactly true	2.83	0.66	−0.346^**^	−0.450^**^	−0.577^**^	(0.92)	
5. Social Support^5^	I know a very close person whose help I can always count on – (1) not true at all to (5) very true	3.84	1.0	−0.225^*^	−0.291^**^	−0.388^**^	0.309^**^	(0.86)

^1^Loneliness aka feelings of loneliness, subjective social isolation was measured with the scale TILS – Three Items Loneliness Scale by Cihlar et al. (67), adapted from Hughes et al. (63). ^2^Anxiety aka fear emotion, negative affect was measured with the scale HADS-D – Hospital Anxiety and Depression Scale-German Version, HADS-A, by Herrmann-Lingen et al. (64). ^3^Depression aka depressive mood or disorder was measured with the scale HADS-D – Hospital Anxiety and Depression Scale-German Version, HADS-D, by Herrmann-Lingen et al. (64). ^4, 5^refer also to social determinants of health was measured with the scale F-SozU K-6 – Perceived Social Support Questionnaire by Kliem et al. (65), and the SWE-General Self-Efficacy Expectancy Scale by Schwarzer and Jerusalem (66). Numbers in the diagonal line are the Cronbach alpha scores of each construct. *p < 0.05; **p < 0.001. According to Cohen (70), the values of the correlation coefficient (*r*) below 0.3 are small, 0.3 – 0.7 are moderate, and above 0.7 are large.

## Results

### Difference between COVID and non-COVID patients in depressive symptomatology

As shown in Table 2 and Table A1, there are no group differences between multimorbid patients hospitalized after a COVID-19 infection and multimorbid patients who were not knowingly infected prior to their clinical stay. A multivariate analysis of variance for joint testing of loneliness, depression, and anxiety showed no significant difference (*F*_(3, 122)_ = 0.255; *p* = 0.86) (Table A1).

Multimorbid patients with a previous COVID-19 infection are not more depressed, anxious, or lonely than multimorbid patients admitted to the clinic for other causes of treatment. This confirms the first hypothesis that an increase in depression and anxiety symptoms in multimorbid patients is not post-COVID specific. An additional analysis of covariance also revealed no differences in mean values regarding the age of the participants between the groups (*b* = −0.01, *t* = −1.354, *p* = 0.178).

### Associations of mental health constraints with loneliness, self-efficacy, and social support

Table 3 shows the interrelationships between mental health constraints (i.e., depression and anxiety symptomatology), loneliness, self-efficacy, and perceived social support in multimorbid patients (hypothesis 2). The results revealed that more mental health constraints (i.e., depression and anxiety symptomatology), are associated with increased loneliness scores (*r*_anxiety_ = 0.496, *p* < 0.001; *r*_depression_ = 0.419, *p* < 0.001), low self-efficacy beliefs (*r*_anxiety_ = −0.450, *p* < 0.001; *r*_depression_ = −0.577, *p* < 0.001), and insufficient perceived social support (*r*_anxiety_ = −0.291, *p* < 0.001; *r*_depression_ = −0.388, *p* < 0.001). All intercorrelations were significant (Table 3).

### Results of mediation and moderation analysis

**Figure 2:**
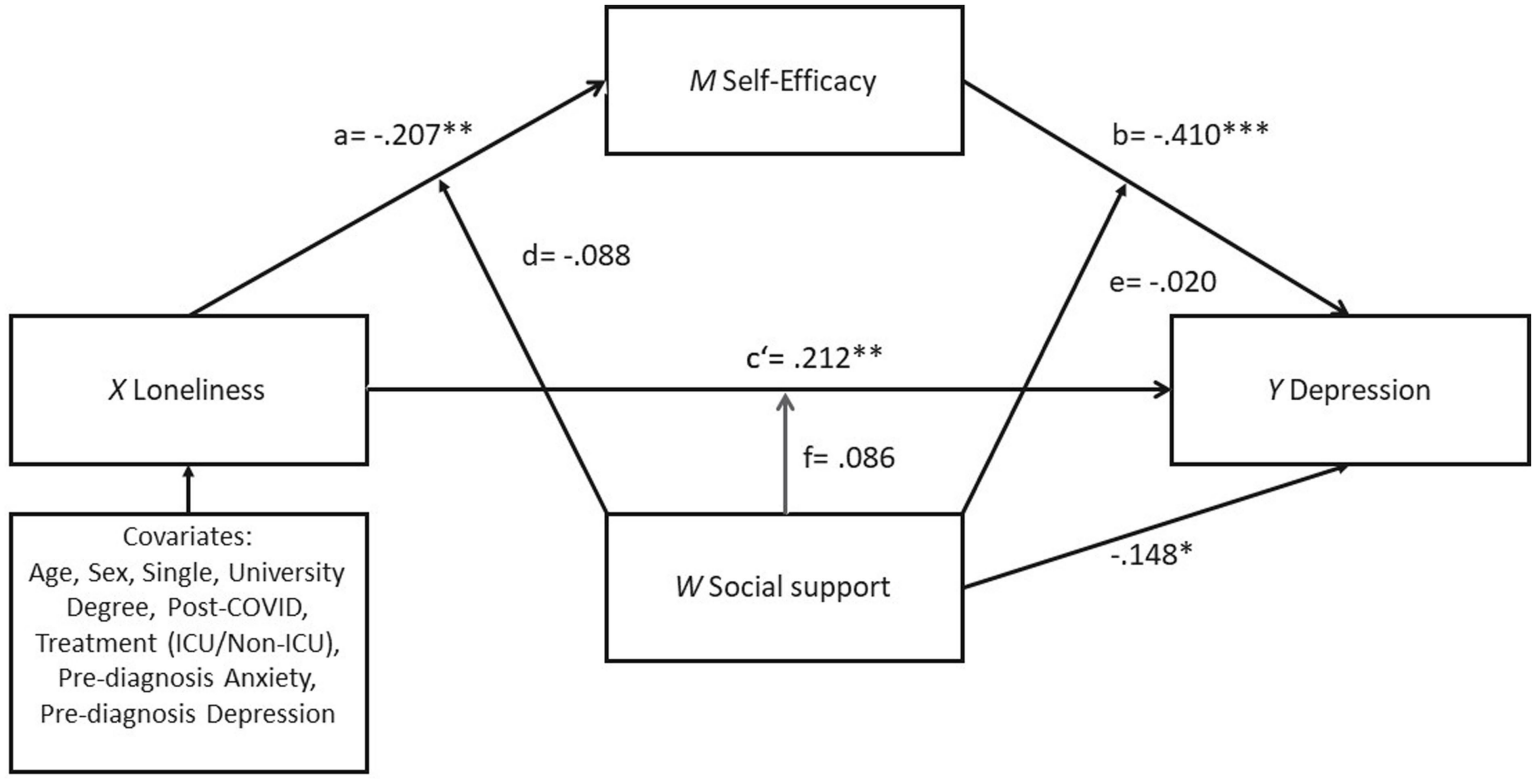
Result of the PROCESS model 59 with a moderated mediation model predicting depression by loneliness with self-efficacy as mediator and social support as moderator. *p < 0.05; **p < 0.01; ***p < 0.001.

The results of the mediation and moderation analysis can be found in Figure 2 and in Appendix (Table A2, A3). For the mediating effect of self-efficacy, as shown in the moderated mediation model (Figure 2), loneliness is significantly associated with self-efficacy (*b* = −0.207, *SE* = 0.077, *p* < 0.01, *R^2^* = 0.305), after controlling for the significant confounding effects of covariates (e.g., age, sex, university degree, and single) (*b* = −0.293, *SE* = 0.109, *p* < 0.01). Self-efficacy was found to be significant negatively associated with depression (*b* = −0.410, *SE* = 0.091, *p* < 0.001). After controlling for the self-efficacy and social support, the association between loneliness and depression achieved the statistical significance (*b* = 0.212, *SE* = 0.064, *p* < 0.01, *R^2^* = 0.519), indicating a partial mediation effect.

For the moderating effect of social support, the results revealed a significant association between perceived social support and depression (*b* = −0.148, *SE* = 0.065, *p* < 0.05), but the interactions of social support with loneliness and self-efficacy were found to be nonsignificant (Path d [loneliness x social support on self-efficacy]: *b* = −0.088, *SE* = 0.106, *p* = 0.411, *R*^2^-change =0.011; Path e [self-efficacy x social support on depression]: *b* = −0.020, *SE* = 0.138 *p* = 0.882, *R*^2^-change =0.000; Path f [loneliness x social support on depression]: *b* = 0.086, *SE* = 0.089 *p* = 0.335, *R^2^*-change =0.006).

**Figure 3:**
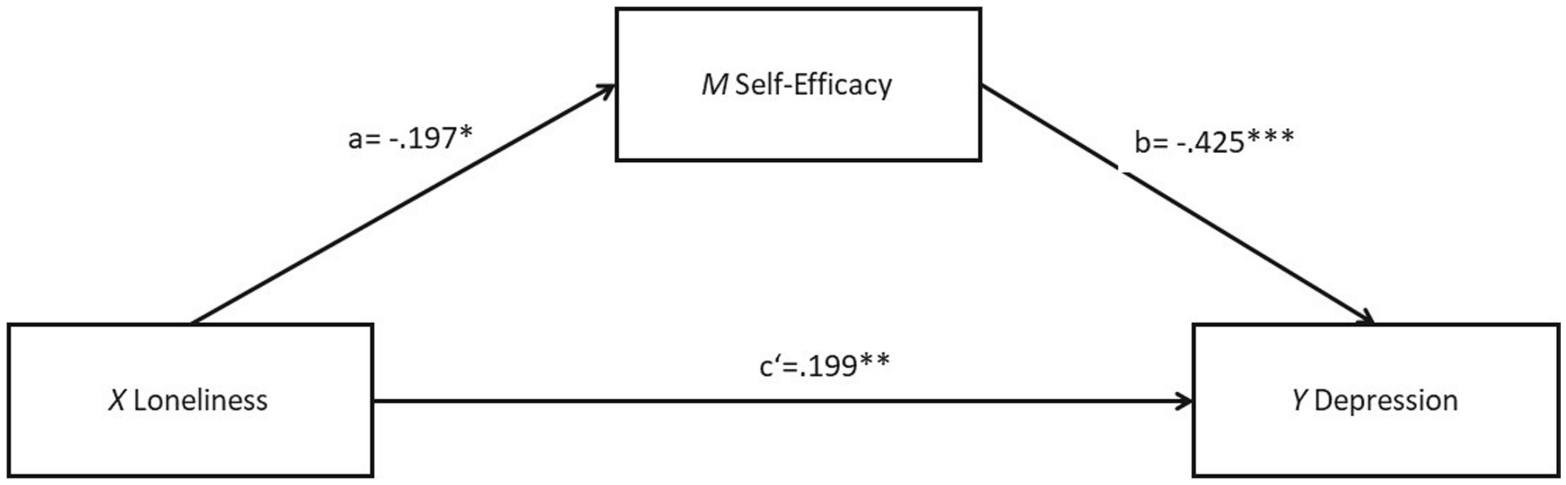
Result of the PROCESS model 4 with a mediation model predicting depression by loneliness with self-efficacy as mediator (social support as moderator left out). *p < 0.05; **p < 0.01; ***p < 0.001.

In addition, a high multicollinearity between the loneliness and social support was identified, with Tolerance = 0.061, VIF = 16.439, and *r* = −0.225. This can lead to less reliable parameter estimates and higher standard errors, making it more difficult to find a significant effect. Therefore, a purely mediation model that excluded the variable of perceived social support was further examined as sensitivity test to validate the robustness of the primary analysis. As shown in Figure 3, loneliness is significantly associated with both self-efficacy (*b* = −0.197, *SE* = 0.075, *p* < 0.05, *R^2^ =* 0.295) and depression (*b* = 0.283, *SE* = 0.062, *p* < 0.001, *R^2^ =* 0.398). A significant association was also found between self-efficacy and depression (*b* = −0.425, *SE* = 0.088, *p* < 0.001). After adding self-efficacy as a mediator, the relationship between loneliness and depression was lessened but still statistically significant (*b* = 0.199, *SE* = 0.065, *p* < 0.01, *R^2^* = 0.508), supporting for the partial mediating effect of self-efficacy. The details for the total effect, indirect effect and direct effect are outlined in Appendix Table A3.

## Discussion

The present cross-sectional study aimed to investigate the mental status of multimorbid patients during hospitalization, focusing on the potential function of loneliness, self-efficacy, and perceived social support for depression and anxiety symptomology.

Firstly, for hypothesis 1, no significant difference in depressive symptomatology was found between multimorbid post-COVID-patients and non-COVID patients, indicating that the hypothesis 1 was supported. This may be because the target group is already more burdened than the general population, which experiences a significant (somatic) health stressor for the first time with post-COVID difficulties. In other words, we face limited variance in the sample of this study and accordingly little difference between the two groups.

Despite prior findings about multimorbid patients being familiar with corresponding life restrictions and poorer health (75), these findings cannot be conclusively clarified in this study and calls for more systematic research with multimorbid patients. Previous studies have found that COVID-19 infection correlated with development of anxiety and depression [e.g., (6, 8)]. However, the samples in these studies have no previous record of multimorbid sickness. In a study conducted using samples with COVID-19 patients, social support explained the prevalence of depressive symptoms among participants (9). Accordingly, investigation of such interrelations is valuable and helpful for design interventions. Taken together, the results supported our proposed hypotheses, demonstrating that multimorbid patients are affected by depression independently of a potential previous infection with COVID-19. This finding was important as with our further analyses we did not have to take the infection status into account when investigating further factors affecting the mental health of multimorbid patients. This implies that we were able to include all patients identified as multimorbid together in the further analyses (correlations, moderated mediation, mediation).

This study also found supporting results regarding hypothesis 2, where high correlations were found between loneliness, self-efficacy, social support and mental health constraints in terms of depression and anxiety. The study found that the more patients reported loneliness, the higher their anxiety and depression scores, the lower their self-efficacy and perceived social support. This finding is in line with previous findings such as that loneliness has repeatedly been found to be associated with depression (21, 25). This finding also provides empirical evidence in favor of social-cognitive theory, emphasizing the interrelationship between self-efficacy, social support and health outcomes.

Also, loneliness has been identified as a mediator variable between other psychological constructs (32), which is in line with the current study findings. Similarly, our findings confirm previous study findings that established a negative correlation between social support and depression (9, 55, 56). This suggests the importance of social support as having a functional network of family and friends to decrease mental health constraints among multimorbid patients. Concretely providing social support directly, helping accompanying people to provide social support and patients to mobilize social support are key components for designing interventions for multimorbid patients (9, 76–79).

The study also utilized moderated mediation analysis to test hypotheses 3 and 4 (i.e., the function of self-efficacy and social support between loneliness and depressive symptoms). Self-efficacy was found to be a mediator between loneliness and depression supporting hypothesis 3. This supports the theoretical assumption of social-cognitive theory and is in line with previous studies that have found that high self-efficacy scores correlate with reduced levels of loneliness and depressive symptoms and better overall health score (25, 44–46). Also, this underlines the importance of strengthening self-efficacy in individuals suffering from loneliness by means of concrete interventions. This can be done by different modes such as giving the option for own mastery experience but also model learning and verbal persuasion (23, 41, 44). However, the interaction between loneliness, self-efficacy and depressive symptoms was a unique contribution of this study: Despite finding an interaction between loneliness, self-efficacy and depressive symptoms, this study did not reveal a significant interaction between perceived social support, loneliness and depression. Social support is not a moderator among loneliness and depression in this study. Therefore, the hypothesis 4 is not supported. Nevertheless, the correlations between loneliness and social support and between social support and depression were significant and demonstrated that social support is directly negatively associated with depression (Table 3). This suggests that social support has a direct importance for the well-being of multimorbid patients in the relation between loneliness and depression and should be considered in further studies, possibly also as a mediator. This conclusion also adds to previous findings by Liu et al. (56), that indicates social support as a mediator between loneliness and depressive symptoms. McAuley et al. (43) demonstrated a strong correlation between the constructs of self-efficacy and social support based on the social-cognitive theory, which is consistent with the correlation results of this study.

The results of this study have several practical implications that provide better interventions for improving the mental health of multimorbid patients. Given the significant associations between loneliness, self-efficacy, and depression, interventions aiming to alleviate depression can take into consideration the aforementioned factors for better impact: Making use of loneliness as a warning signal, pressure to change and target for evaluations, interventions to prevent mental health problems might become more manageable (28). Identifying at risk patients by means of loneliness might be easy with short symptom checklists [e.g., TILS – Three Items Loneliness Scale by Hughes et al. (63)] or asking corresponding questions. Addressing loneliness can be done by typical tools like connecting patients with each other, helping interaction with relatives and friends or also improving communication with health professionals could help (9, 76–79). Moreover, healthcare providers may consider implementing interventions to enhance self-efficacy, such as cognitive-behavioral therapy, which has been shown to improve self-efficacy and reduce depressive symptoms at an early point in time (39). Additionally, strategies that aim to improve social connectedness and reduce social isolation could be beneficial for overall mental well-being. Interventions aiming at mental health in elderly multimorbid patients should address loneliness, self-efficacy, and social support in addition to only caring for their physical, cognitive, and mental health. As lifestyles approaches have potential to also overcome loneliness, strengthen self-efficacy and social support, they should be employed accordingly. The current study found that social support has a significant negative correlation with depressive symptomology. Hence, future intervention should consider providing multimorbid patients with social support to prevent the development of mental health issues. Lifestyle as general social-cognitive factors such as self-efficacy and social support was clearly demonstrated as effective. On basis of previous evidence for behaviors such as caring activities and social activities (2), interventions should integrate them as well, especially in individuals facing decline in later life: especially in hospitalized elderly multimorbid patients with and without known COVID-19 infection.

As indicated above, the results of this study are a possible basis for specific interventions: e.g., interventions could have the promotion of health and physical activity goals as a focus, or specifically address the expansion of the social action radius to enable patients to pursue a more socially active lifestyle. Part of the interventions should be psychoeducation about the health benefits for soma and psyche when physical activity, perceived social support and belief in one’s own self efficacy are strengthened despite multimorbidity. Likewise, identifying and activating existing resources that the patient already contributes should be integrated into interventions.

According to the National Health Service (NHS) (80), the social prescribing approach is cross-age and particularly suitable for the non-medical support of people who are socially isolated or lonely, suffer from one or more chronic illnesses and mental health problems, and whose well-being is impaired. Link workers can advise patients, identify their needs and matching resources, the patients are to be directed to suitable offers in the community. Taking this into account could improve active coping of patients suffering from loneliness. Additionally, strategies that aim to improve social connectedness and reduce social isolation could be beneficial for overall mental well-being. Future research should test such prevention strategies and interventions not only in a longitudinal design but also with experimental methodology. This leads us to reflect on the limitations of the current study and what to suggest for future research, which will be described in the following section.

### Limitations and suggestions for future research

The study faces several limitations pertaining to different aspects of the design, sample size and results. We only focused on two aspects of mental health namely anxiety and depression. Thus, future studies could also integrate other aspects of mental health such as general emotional, psychological, and social well-being and not only anxiety and depressive symptoms. The cross-sectional design of this study does not allow conclusions to be drawn about causality. A causal relationship between loneliness and depression should therefore be further explored. Hence, longitudinal studies are needed to confirm the directionality of the relationships found in the study. Additionally, the sample size was restricted to those who did not have language barriers that might have impacted the study, which can also impact the generalizability of the results. Accordingly, future studies should aim to recruit a more diverse sample. For instance, providing the questionnaire in different languages and assisting the patients in filling it out with people from their cultural background could be a great advantage. Moreover, replicating this study in other cultures and countries such as Asia are needed as the current findings might be specific for Germany or individualistic cultures only. Testing the findings in collectivistic cultures and especially the functions of self-efficacy and social support is needed to help patients world-wide. In subsequent studies, the Social Determinants of Health (SDH) as defined by the World Health Organization (WHO) should be included in the surveys and statistical analyses. According to World Health Organization (WHO) (3), the SDH characterize conditions under which people are born, live, work, and spend their daily lives. These factors are non-medical and yet play a significant role in forming a person’s physical and mental health in both positive and negative ways. The following Social Determinants of Health have been defined by World Health Organization (WHO) (3): income and social protection, education, unemployment and job insecurity, working conditions. Likewise, food insecurity, housing, basic services and environment, early childhood development, social inclusion, non-discrimination, and structural conflicts were included. Access to affordable health services of adequate quality was also factored into the definition.

There was also high multicollinearity between loneliness and social support constructs, which may have impacted the reliability of the moderated mediation analysis. Further research is warranted to explore these relationships more thoroughly. Finally, the results were exclusively based on patients’ self-assessments, which might raise questions regarding the impact of social desirability on the results. Additionally, the quality and representativeness of self-assessment by means of questionnaires may differ during hospital stays compared to a more familiar and less stressful environment. Data collected from multimorbid patients in their home setting could potentially provide different results. Ethnographic observations and objective measures of the health status should add to such improvements of the methodology. Lastly, as there is no pre-pandemic data available for comparing results, the sample can only be evaluated in the context of the pandemic. Replication is warranted with data post-pandemic, ideally also taking post-COVID symptoms into account.

Future approaches could also use the data presented in this study as a baseline for research on the correlation predictors of depression among multimorbid patients and should aim to replicate the findings in general. Future studies can also attempt to validate the data using samples from patients across the world who suffered from other illnesses to investigate whether the findings can generalize across cultures. Additionally, researchers can utilize longitudinal approach to better understand the influence of loneliness, self-efficacy, and social support on multimorbid patients and how symptoms develop across time. Intervention programs are required to provide better understanding of the best ways to mitigate psychological constraints.

This study also provides an important insight for future researchers regarding the interrelation of loneliness with depressive symptomology in multimorbid patients regardless of the COVID-19 infection status. This is also in correlation with previous studies that found significant longitudinal impact between loneliness and depression (21, 30, 31), especially those diagnosed with cancer (81).

## Conclusion

This study found no interrelation between COVID-19 infection and depressive symptoms in elderly multimorbid patients. Additionally, the study found no moderation relationship between perceived social support, loneliness, and depression. This suggests that perceived social support and loneliness are both associated with the level of depression in multimorbid patients. Self-efficacy, however, was found to mediate the relationship between loneliness and depression. These findings emphasize the importance of tackling loneliness, self-efficacy, and perceived social support when attempting to alleviate depression symptoms in elderly multimorbid patients. The study also highlights the importance of continued care for elderly multimorbid patients in principle, not only during times of scarce resources such as during a pandemic or for other reasons that limit access to care. As these patients generally require effective health care to prevent them from the development of sustainable mental health constraints.

Especially, because older people are at risk of developing multimorbid conditions, which can lead to various physical and mental health problems including cognitive decline in late life if left untreated. Previous studies have found several factors that can promote the mental health of aging adults, including spiritual comfort services (82), and tailored physical activity programs (83, 84). This study complements previous findings in highlighting the importance of self-efficacy, and social support as lifestyle factors. When aiming at prevention and treatment of depression and subjective cognitive decline in multimorbid patients, these findings can help addressing imperative targets.

## Data availability statement

The raw data supporting the conclusions of this article will be made available by the authors, without undue reservation.

## Ethics statement

The human studies were approved by the Ethics Committee of Constructor University (formerly Jacobs University Bremen), Bremen, Germany (Application No: 2020_06). The studies were conducted in accordance with the local legislation and institutional requirements. The participants provided their written informed consent to participate in this study.

## Author contributions

AR conceived and directed the research and wrote the first manuscript and organized the database and performed the statistical analysis. AR, SL, and HA-S wrote parts of the manuscript, revised and completed the manuscript, and were jointly responsible for the final content with AR, WL, and YD. AR and SL contributed to the conception and design of the study. SL monitored the progress of the study. WL and YD contributed to revisions and content additions to the manuscript, and read and approved the submitted version. All authors contributed to the article and approved the submitted version.

## Figures and Tables

**Table:** 

Term	Related terms	Definition
Loneliness	Feelings of loneliness, subjective social isolation, involuntary isolation, involuntary solitude	Characterization of loneliness as perceived social isolation, emotional loneliness refers to the absence of a close bond such as the (spouse) partner, family members, social loneliness describes the absence of social belonging to a network (71). Loneliness corresponds to a discrepancy between a person's preferred and actual social relationships (72, 85). Feeling lonely does not necessarily mean being alone, as people can also feel lonely in a group of people (29)
Self-Efficacy	Self-belief, self-confidence, self-assuredness, conviction	Perceived self-efficacy is defined as the belief of individuals that they can successfully perform a certain behavior in certain situations (36).
Social Support	Social well-being, social interaction	Social support refers to the subjective or anticipated perception of support from a person's social network. This subjective assessment is based on past and present social interactions (73).
