# Supplementary material for: Loneliness and depression in older adults with multimorbidity: the role of self-efficacy and social support
Source: Front Psychiatry. 2023 Oct 30;14:1232067. doi: 10.3389/fpsyt.2023.1232067 (PMC10642299; doi:10.3389/fpsyt.2023.1232067)
Supplement: Supplementary file 1 [file Data_Sheet_1.PDF]

CONSTRUCTOR UNIVERSITY

Annika Roskoschinski
PhD Fellow
Department of Health Psychology
and Behavioral Medicine
aroskoschinski@constructor.universit
y

Constructor University Bremen gGmbH · Campus Ring 1 · 28759 Bremen · Germany

www.constructor.university

Frontiers in Psychiatry
To the Reviewers and Editor

Bremen, 10/05/2023

Revision Letter on Ms “Loneliness and depression in older adults with multimorbidity: The role of self-efficacy and social support”

Annika Roskoschinski, Wei Liang, Yanping Duan, Hayl Al-Salehi and Sonia Lippke*

Original Research, Front. Psychiatry - Public Mental Health

Received on: 31 May 2023, Edited by: Joseph Gallo

Manuscript ID: 1232067

Reviewer 3: finalized

Reviewer 4: 5 October 2023

Dear Editor and Reviewer,

thank you very much for your further feedback, which contributes to the improvement of the quality and clarification of our manuscript, which pleases us very much. We have conscientiously incorporated all responses and hope that the manuscript will now meet with your appreciation.

Hereby, we are resubmitting our manuscript entitled "Loneliness and depression in older adults with multimorbidity: the role of self-efficacy and social support" after careful revision in response to the reviewers' comments.

We thank you for considering our manuscript for publication in Frontiers Psychiatry - Public Mental Health and look forward to the manuscript being accepted for publication soon.

Yours sincerely,

Annika Roskoschinski on behalf of all Authors

CONSTRUCTOR UNIVERSITY

Reviewer 4 | 03 Oct 2023 | 17:11

#3

Overview

The manuscript reviewed is a revision of report of a study conducted that explored loneliness, self-efficacy, social support, and depression within the context of multiple co-morbidities and COVID-19 infection. The study has value and offers general clinical treatment recommendations to address self-efficacy, social support, and loneliness.

This revision carefully addresses the comments provided previously. Although they have tempered their desire to find a causal relationship between the variables in this cross-sectional study, they continue to push the boundaries and insert language that suggests a causal relationship exists.

Following a few more changes that would strengthen, clarify, and make the manuscript more accurately reflect what a cross sectional study can claim.

Page 4, line 82: I believe when you say “scores” you mean “symptoms”

Page 11, line 263: You indicate that patients age 18 and older were recruited, but earlier, you indicated that the study population were participants age 65 and older.

Page 25, line 606-7: Cross-sectional designs do not allow one to establish causal relationships. This sentence must be corrected. The next sentence must also be corrected. The word “The” at the beginning of the sentence is incorrectly used. It presumes that a causal relationship was found when it was not.

Page 28, line 686: Instead of “both contribute individually to” I believe that “are both associated with” would be more accurate.

Authors Response

We are very keen to implement your comments and changes so that the content of the manuscript is even clearer and more sharply defined. This is also a concern for us, and we hope to have pleased you with the changes now made and to have clearly described that we do not assume a causal relationship with the cross-sectional study, but merely want to encourage further studies to investigate the associations we describe in more detail.

Regarding the Overview:

We thank you very much for your patience and valuable feedback to further improve our manuscript.

Specifically, we have implemented your changes as follows:

REVIEWERS COMMENT: Regarding Comment on Page 4, line 82: I believe when you say “scores” you mean “symptoms”:

OUR RESPONSE: We apologize. Yes, we meant "symptoms" and corrected that.

It now reads, p. 4, line 81: "They concluded that during the course (approximately 2 months), an increased number of COVID-19 symptoms was associated with clinically significant increased scores for anxiety 22.4% (n = 201) and depression 26.2% (n = 235) and recommended monitoring the development of these symptoms after discharge from COVID-19 treatment."

We also checked all other instances where “scores” was used to make sure that this is correct. Whenever “scores” is used, it represents the mean level.

Accordingly, we have taken the option of also pointing out again at the end of the introduction, page 9, line 211, that we are examining associations to clarify this.

This now reads, p. 9, line 211: "In addition, this study aimed to examine *associations* and the relationship between loneliness and depression, as well as the mediating role of self-efficacy and moderating role of perceived social support in the aforementioned association in older adults with multimorbidity."

We have also emphasized once again in the "Method" section at the beginning that this is a cross-sectional study examining associations.

It now reads, p. 10, line 227-229: "To test the hypotheses, a cross-sectional study with one measurement point was conducted to examine associations between the variables loneliness and depression as well as a mediating and moderating role of self-efficacy and social support."

REVIEWERS COMMENT: Regarding Comment on Page 11, line 263: You indicate that patients age 18 and older were recruited, but earlier, you indicated that the study population were participants age 65 and older:

OUR RESPONSE: The line "patients 18 years and older" only meant that patients were of legal age. The department in which the data were collected is specifically the Unit of Physical Medicine and Geriatrics. Because this is confusing, we have deleted this part. Thus, it now reads, p. 11, line 253: „Patients who were multimorbid, previously diagnosed with COVID-19, and multimorbid patients without previous COVID-19 infection were recruited for the study."

REVIEWERS COMMENT: Regarding Comment on Page 25, line 606-7: Cross-sectional designs do not allow one to establish causal relationships. This sentence must be corrected. The next sentence must also be corrected. The word "The" at the beginning of the sentence is incorrectly used. It presumes that a causal relationship was found when it was not.

OUR RESPONSE: We have revised the text sections according to your guidance and proofread the manuscript again to ensure that the impression of the claim of a causal relationship is excluded.

It now reads, p. 21, line 499-501: " The cross-sectional design of this study does not allow conclusions to be drawn about causality. A causal relationship between loneliness and depression should therefore be further explored."

REVIEWERS COMMENT: Regarding Comment on Page 28, line 686: Instead of "both contribute individually to" I believe that "are both associated with" would be more accurate.

OUR RESPONSE: Thank you, we changed that to describe it accurately.

It now reads, p. 23, lines 554-555: "This suggests that perceived social support and loneliness are both associated with the level of depression in multimorbid patients."

We apologize for the misunderstanding and typos, and accordingly also checked the whole manuscript again. We performed all needed amendments and hope that these changes facilitate the acceptance of our paper.
